# Von Willebrand Factor, Factor VIII, and Other Acute Phase Reactants as Biomarkers of Inflammation and Endothelial Dysfunction in Chronic Graft-Versus-Host Disease

**DOI:** 10.3389/fimmu.2021.676756

**Published:** 2021-04-30

**Authors:** Antonela Lelas, Hildegard Theresia Greinix, Daniel Wolff, Günther Eissner, Steven Zivko Pavletic, Drazen Pulanic

**Affiliations:** ^1^ Division of Hematology, Department of Internal Medicine, University Hospital Centre Zagreb, Zagreb, Croatia; ^2^ Division of Hematology, Medical University of Graz, Graz, Austria; ^3^ Department of Internal Medicine III, University Hospital Regensburg, Regensburg, Germany; ^4^ Systems Biology Ireland, School of Medicine, Conway Institute, University College Dublin, Dublin, Ireland; ^5^ Center for Cancer Research, National Cancer Institute, National Institutes of Health, Bethesda, MD, United States; ^6^ School of Medicine, University of Zagreb, Zagreb, Croatia

**Keywords:** endothelial dysfunction, inflammation, factor VIII, Von Willeband factor, chronic graft-versus-host disease (cGvHD), allogeneic hematopoietic stem cell transplantation

## Abstract

Chronic graft-versus-host disease (cGvHD) is an immune mediated late complication of allogeneic hematopoietic stem cell transplantation (alloHSCT). Discovery of adequate biomarkers could identify high-risk patients and provide an effective pre-emptive intervention or early modification of therapeutic strategy, thus reducing prevalence and severity of the disease among long-term survivors of alloHSCT. Inflammation, endothelial injury, and endothelial dysfunction are involved in cGvHD development. Altered levels of acute phase reactants have shown a strong correlation with the activity of several immune mediated disorders and are routinely used in clinical practice. Since elevated von Willebrand factor (VWF) and factor VIII (FVIII) levels have been described as acute phase reactants that may indicate endothelial dysfunction and inflammation in different settings, including chronic autoimmune diseases, they could serve as potential candidate biomarkers of cGvHD. In this review we focused on reported data regarding VWF and FVIII as well as other markers of inflammation and endothelial dysfunction, evaluating their potential role in cGvHD.

## Introduction

Chronic graft-versus-host disease (cGvHD) is an iatrogenic, immune mediated complication of allogeneic hematopoietic stem cell transplantation (alloHSCT) with clinical features resembling aspects of several autoimmune disorders. With its high incidence of approximately 50% (1) and mortality of approximately 25% (2), cGvHD is a major burden on quality of life and long-term survival following alloHSCT. Considering the fact that improvement of transplantation techniques allowed an increased number of patients to undergo alloHSCT and resulted in longer survival, the future incidence of cGvHD is expected to become even higher (1). Despite tremendous efforts of the scientific community put into research of cGvHD biology, a significant breakthrough in treatment strategies has not been achieved yet. One of the major obstacles for further progress is the absence of biomarkers suitable for clinical use. An ideal biomarker would be a specific agent that is active early in cGvHD development and that reliably identifies high-risk patients. This biomarker would allow developing of effective pre-emptive intervention, thus reducing incidence and severity of cGvHD. Furthermore, a specific marker that could provide earlier and more accurate initial assessment of disease intensity, or detect therapeutic response before improvement or worsening of clinical manifestations, could revolutionize the therapeutic approach. For all these reasons, along with the search for an effective treatment, the search for a biomarker that has satisfactory specificity and sensitivity has become one of the top priorities of researchers and clinicians (3, 4). However, the elusive etiology of cGvHD significantly hinders this search so far, especially when masked by multifarious impacts that can modulate course of illness. It is widely known that both inflammation and endothelial injury persist long after alloHSCT, and are involved in cGvHD development (5–8). Von Willebrand factor (VWF) and factor VIII (FVIII) are coagulation factors, as well as acute phase reactants indicating endothelial dysfunction and inflammation in different settings. Therefore, they could serve as potentially interesting candidate biomarkers of cGvHD. In this review we focused on reported data regarding VWF and FVIII, and other markers of inflammation and endothelial dysfunction, evaluating their potential role in cGvHD.

## Von Willebrand Factor and Factor VIII

### General Considerations

Von Willebrand factor and FVIII are widely known as coagulation factors that play an important role in hemostasis. Beside the critical role of VWF in “platelet plug” formation (bridging molecule for platelet adhesion and promoting platelet aggregation) at sites of vascular injury, it also serves as a carrier of FVIII, protecting it from proteolytic degradation and transporting it to sites of injury, thus enhancing fibrin formation (9). A detailed overview of VWF and FVIII physiology is shown in **Table 1**. In this review we will focus on the fact that both VWF and FVIII are considered positive acute phase reactants in settings of inflammation and endothelial activation (11).

**Table 1:** General information about VWF and FVIII (9, 10).

	VWF	FVIII
Structure	multimeric glycoprotein	sialoglycoprotein
Size	up to 20,000 kDa (monomer 250 kDa)	270 kDa
Plasma concentration	10 μg/ml (~35 nM)	200 ng/ml (~0.8 nM)
Role	platelet adhesion (activation + recruitment) carrier for and stabilization of FVIII	blood coagulation - thrombin generation and fibrin clot formation
Origin	megakaryocytes vascular endothelial cells	mostly hepatic sinusoidal endothelial cells, but also endothelial cells of other tissues (lungs, lymphoid, venules)
Half-life in circulation	~12 (9-15) hours	1-2 h as free molecule 8-12 h in a noncovalent complex with VWF
Clearance	mostly together as a complex through macrophages from liver and spleen	
Gene	VWF on chromosome 12	F8 on X chromosome

VWF, von Willebrand factor; FVIII, factor VIII; kDa, kilodalton; μg/ml, micrograms per milliliter; ng/ml, nanogram per milliliter; nM, nanomolar.

Although synthesis regulation of VWF and FVIII is yet to be completely elucidated, it seems that different endothelial cells produce each of them, with an additional production of VWF by megakaryocytes. While most of VWF is synthesized in endothelium of larger extrahepatic vessels, FVIII is synthesized mostly in hepatic sinusoidal endothelial cells. Interestingly, murine models have shown predominant distribution of VWF mRNA in lung and brain, indicating that VWF is differently expressed in different tissues (12). It should be emphasized that VWF and FVIII are the only coagulation factors that are not being produced by liver hepatocytes. Upon synthesis VWF is stored in specialized organelles; in smaller quantities as α granules of platelets, but mostly as Weibel-Palade bodies in vascular endothelial cells. Since endothelium represents the first line of defense from noxious stimuli, its activation caused by vascular injury of any etiology, results in secretion of VWF from Weibel-Palade bodies into the plasma. Secretion can be provoked by different inflammatory mediators released from resident immune cells, mostly tissue macrophages (13), and it results not only in direct adhesive interaction of VWF with leukocytes and trans-endothelial migration (14), but also in stimulation of other proinflammatory pathways such as complement and neutrophil extracellular traps (15). Moreover, proinflammatory agents, such as P-selectin are also released from Weibel-Palade bodies (16) further supporting the inflammatory response.

While VWF is well-recognized as the carrier of FVIII in plasma and protector of its degradation, the possible impact of FVIII on VWF plasma levels is poorly resolved. Some studies described a synergistic role of FVIII and platelets on the cleavage of VWF multimers by a disintegrin and metalloproteinase with ADAMTS13 (a thrombospondin type 1 motif, member 13) (17), while study with FVIII deficient mouse model revealed increased VWF content in the liver endothelium and increased VWF plasma levels, which was associated with hepatic low-grade inflammation (18).

### Clinical Biomarker Utility

The clinical utility of VWF and FVIII as biomarkers has been questioned due to a possible influence by many extrinsic patient related parameters, such as age, body weight, ABO antigen status, diet, smoking, ethnicity, and even exercise (10, 19–21). However, possible benefits of such a relatively cost-effective and non-invasive diagnostic tool encouraged extensive research in the field of cardiovascular diseases, resulting in clear distinction of patients at risk for major adverse cardiovascular events (22–24). Significant elevation of both biomarkers has been described in different states of acute stress, and lately a lot of attention has been given to the investigation of their role in connection between inflammation and procoagulant state caused by coronavirus 2019 (SARS-CoV-2) infection (25, 26). Elevation of VWF and FVIII was also reported in many long-lasting clinical conditions, including chronic autoimmune rheumatic diseases, pregnancy, malignancy, hyperthyroidism, hyperglycemia, hypertriglyceridemia, liver and renal disease (19, 27, 28). All these findings directed current research of VWF and FVIII toward elucidation of association between hemostasis, thrombosis, endothelial injury, and inflammation in a process jointly named as “thromboinflammation”. Although increase of these coagulation factors has been described in many vasculopathies and other states of hypoxia, this review mainly focusses on autoimmune diseases that clinically resemble cGvHD. Available knowledge of biomarker utility in different settings regarding VWF is summarized in **Table 2**, while one regarding FVIII is shown in **Table 3**. It is interesting to note that both VWF and FVIII level increase correlates with activity of autoimmune connective tissue diseases. In systemic sclerosis and systemic lupus erythematosus it has shown to be predictive of pulmonary involvement (31), which can be connected to localized endothelial synthesis of these factors. Pathogenesis of both of these diseases includes inflammation and microvascular changes that lead to hypoxia and, consequently, fibrosis which is similar as in cGvHD. From these studies it can be concluded that inflammation caused by autoimmune disease is a consequence of endothelial injury. However, a detailed analysis of the biomarker potential regarding VWF and FVIII, including their sensitivity and specificity, has not been performed yet.

**Table 2:** Reported biomarker potential of elevated VWF levels in different diseases/conditions.

Disease	Result	References
Systemic sclerosis/scleroderma	correlates with activity of disease	(29)
	levels proportional to severity and involvement	(30)
	predictive of pulmonary hypertension	(31)
Systemic lupus erythematosus	correlates with activity of disease, increase proportional to level of inflammation	(32)
	predicts pulmonary arterial hypertension	(31)
Rheumatoid arthritis (RA)	correlates with activity of disease	(33)
	increased in vasculitic form of RA	(34)
Wegener`s granulomatosis	correlates with activity of disease	(35)
Behçet’s disease	correlates with activity of disease	(36)
Raynaud`s phenomenon	correlates with activity of disease	(37)
Hemolytic uremic syndrome	correlates with severity of disease	(38)
Henoch-Schönlein purpura	correlates with severity of disease	(39)
Coronary artery disease	after admission - risk of adverse cardiovascular outcome and death during one-year follow-up	(40, 41)
	higher vWF in acute coronary syndrome than in stable angina pectoris, but in angina pectoris connected to higher coronary plaque burden	(41)
	increased coronary plaque burden, marker of residual cardiovascular risk after statin therapy	(42)
	poor recanalization and worse outcome after thrombolysis for acute myocardial infarction	(43)
Arterial hypertension	predictive for appearance or progression of atherosclerosis	(44)
Atrial fibrillation	risk of major adverse cardiovascular event, bleeding and all-cause mortality, may discriminate patients at risk	(45–49)
Heart failure	severity of right heart failure and related liver dysfunction, prognostic for all-cause mortality in adults with congenital heart disease	(50)
	prediction of short term adverse events/outcome	(51)
Cerebrovascular insult	mortality, morbidity, severity, recurrence	(52, 53)
	outcome, risk stratification	(54, 55)
Diabetes mellitus II	risk of cardiovascular diseases	(56, 57)
Carotid stenosis	predictive for major cardiovascular events	(58)
Chronic liver disease	predictor of clinically significant/severe portal hypertension and mortality in patients with liver cirrhosis	(59–61)
	short-term mortality among patients with acute-on-chronic liver failure	(62, 63)
	development of hepatopulmonary syndrome	(64)
Hepatitis B and C	marker for liver fibrosis and prediction of hepatocellular carcinoma development	(65, 66)
Hepatocellular carcinoma	higher post-operative levels correlate to early relapse in 2 year follow up	(67)
	higher preoperative levels correlate to early postoperative liver dysfunction, morbidity and mortality	(68, 69)
Liver transplantation	increase risk of re-transplantation, independent prognostic factor for re-transplantation-free survival	(70)
	increased levels of VWF among patients who died within three months of waiting on list for orthotopic liver transplantation, significant mortality risk stratification	(71)
Breast cancer	preoperative - predict relapse	(72)
Cancer	venous thromboembolism	(73)
	biomarker for early detection of lung adenocarcinoma among patients with type II diabetes mellitus, outcome	(74, 75)
Kaposi sarcoma	activity, but not for the extent of disease	(76)
AL amyloidosis	poor outcome	(77)
Pulmonary hypertension	prognostic	(78)
Acute lung injury/ Acute respiratory distress syndrome	predicts outcome among adults and children, regardless of sepsis or protective ventilator strategy	(79, 80)
	in meta-analysis not predictive of mortality	(81)
Asthma	VWF propeptide – severity and airway structural change measured by MRI but not by CT	(82)
COPD	mortality, different forms of VWF are characteristic for different phenotypes	(83, 84)
Sepsis	outcome in severe infections	(85)
	acute lung injury in non-pulmonary sepsis	(86)
	questionable: predictive of mortality *vs* no predictive value in other study	(87, 88)
Viruses	Sudan virus, predicts adverse outcome	(89)
	COVID-19 – mortality	(25, 26)
Preeclampsia	diagnostic	(90)

VWF, von Willebrand factor; RA, rheumatoid arthritis; AL amyloidosis, light chain amyloidosis; MRI, magnetic resonance imaging; CT, computed tomography; COPD, chronic obstructive pulmonary disease; COVID-19, coronavirus disease 2019.

**Table 3:** Reported biomarker potential of elevated FVIII levels in different diseases/conditions.

Disease	Result	References
Connective tissue diseases	correlates with activity of disease	(91)
Behçet’s disease	correlates with activity of disease	(36)
Raynaud`s phenomenon	correlates with activity of disease	(37)
Hemolytic uremic syndrome	correlates with severity of disease	(38)
Renal transplantation	predicts vascular rejection	(92)
Cerebrovascular insult	outcome, risk stratification	(54, 55)
Venous thromboembolism	recurrence in idiopathic cases	(93)
	predicts patients at risk after anticoagulation withdrawal	(94)
Carcinoma	improves diagnostic accuracy of pancreatic ductal adenocarcinoma	(95)
	prognostic for breast cancer outcome	(96)
	predictive of thromboembolism among cancer patients	(97, 98)
Pregnancy	recurrent early pregnancy loss	(99)

FVIII, factor VIII.

### VWF and FVIII in a Post-alloHSCT Setting

Despite the fact that increase of VWF and FVIII can also be caused by hematologic malignancies (100, 101), chemotherapy or autologous HSCT, their levels following alloHSCT are significantly more prominent (102, 103). It is considered that such increase in a post-alloHSCT setting reflects endothelial injury in addition to acute phase reaction. Endothelium is the first-line barrier against various transplant related toxic effects including cytostatic therapy, radiation, immunosuppression, and alloreactivity. Increase of VWF has already shown prognostic value for acute GvHD (aGvHD) development (104). Moreover, it has been reported that elevated levels of VWF and FVIII among patients diagnosed with aGvHD strongly correlate with endothelial activation but their role in aGvHD biology has not yet been defined (102, 105–108). Such findings suggest that the endothelium has a pathophysiological role in aGvHD development which goes beyond cytokine-mediated cell-to-cell communication. Interestingly, an increase of VWF level was proposed as a diagnostic marker of cGvHD among alloHSCT long-term survivors also (108), with its potential utility as sensitive biomarker of cGvHD activity (109). These reports, though, had a limited number of subjects and lacking methodology. However, recent preliminary results of a prospective study on a well-defined cohort of alloHSCT patients conducted by Pulanic at al. showed significantly higher levels of VWF and FVIII in the cGvHD group in comparison to patients who did not develop cGvHD (110). Further analysis revealed a close association between cGvHD activity and elevated levels of VWF, suggesting a potential causal relationship. It should also be noticed that thromboembolic events, including venous thromboembolism, have been associated with presence of both acute and chronic GvHD even 50 months after alloHSCT (111). Such observations could suggest a potential role of inflammation induced increase of coagulation factors FVIII and VWF in the pathogenesis of these events. Additionally, elevated levels of VWF were described as predictive of poor outcome for some vascular endothelial syndromes such as transplant-associated thrombotic microangiopathy (112, 113), and FVIII seems to be involved in the pathophysiology of sinusoidal obstruction syndrome/veno-occlusive disease (SOS/VOD) after alloHSCT (114). The prognostic role of FVIII and VWF plasma levels in SOS/VOD has been investigated, but the results were too ambiguous for clinical use (115).

## Chronic Graft-Versus-Host Disease and Markers of Inflammation and Endothelial Dysfunction 

The term acute-phase reactants (APRs) relates to all markers whose measured plasma concentrations increase or decrease by at least 25% during inflammation or tissue injury (116). Most of APRs are proteins produced by the liver. Their production is regulated by cytokines like interleukin-2 (IL-2), tumor necrosis factor alpha (TNF-α) and interferon gamma (IFNγ) which are being produced by immune cells during the inflammation process. As already mentioned earlier, inflammation and endothelial injury are closely connected. Also, both processes have been described in the cGvHD setting, although less extensively studied than in aGVHD. The most comprehensive study addressing inflammation in cGvHD was performed by Grkovic and NCI group who investigated clinical laboratory markers of inflammation as determinants of cGvHD activity and severity (6). The authors proposed that reactive thrombocytosis, mediated by interleukin-6 (IL-6), might contribute to the pathogenesis of transforming growth factor beta (TGF-β) and platelet-derived growth factor (PDGF)-induced fibrosis and vascular thickening. Namely, though thrombocytopenia measured at time of diagnosis of cGvHD is among the strongest predictors of poor survival across many studies (117), this study has shown an association of increased platelets with cGvHD activity and with more severe skin and joint/fascia cGvHD involvement over a longer follow-up period after cGvHD presentation (6). This is in line with other studies identifying platelets as key regulators of the inflammatory response (118). In addition to that, strong association of low platelet counts at time of diagnosis of cGvHD and poor survival could be partly due to development of microangiopathic hemolytic anemia (defined by appearance of schistocytes in peripheral blood smears and thrombocytopenia) in some cases, and impaired splenic function may cause thrombocytopenia in others (117).

Endothelial injury caused by inflammation, on the other hand, has not been extensively studied in cGvHD so far. However, an increase of vascular inflammation markers, such as endothelin-1, gelsolin, and anti-LG3 (autoantibodies against perlecan/LG3), was observed, confirming thus important role of endothelial injury and inflammation in the onset of cGvHD (119). Of note, endothelial injury is usually characterized by induction of proinflammatory cytokines (TNF-α, IL-6, IL-10, IL-1β, etc.) and by decreased levels of anti-inflammatory cytokines (TGF-β, IL-15), increased release of coagulation factors (VWF and FVIII), and overexpression of soluble and membrane-bound adhesion molecules (ICAM-1 (intercellular adhesion molecule 1), VCAM-1 (vascular cell adhesion protein 1), E-selectin) (120, 121). Interestingly, in contrast to multitude reports in aGvHD, there is no cGvHD data available even on basic hemostatic APRs, such as fibrinogen, D-dimer, thrombomodulin, and plasminogen activator inhibitor (PAI-1), a well-known marker of endothelial damage in SOS/VOD. Moreover, although inadequate activation of natural anticoagulants has been reported among patients following HSCT (107), no studies with longer follow-ups, describing changes among cGvHD patients in particular, were found. Aside from hemostatic APRs, great progress in omnics technique resulted in a number of novel potential cGvHD biomarkers, further reflecting the complex biology of cGvHD. For example, an interesting study with a panel of four markers (C-X-C motif chemokine ligand 9 (CXCL9), suppression of tumorigenicity 2 (ST2), osteopontin (OPN) and matrix metalloproteinase-3 (MMP3) measured 100 days after alloHSCT showed predictability for cGvHD development (122). The most important APRs and markers of endothelial dysfunction that have been investigated as potential cGvHD biomarkers are summarized in **Table 4**. However, it has to be stated that there is lack of standardized methods to separate inflammation in general from endothelial injury.

**Table 4:** Acute phase reactants and markers of endothelial dysfunction in cGvHD.

Inflammation markers	Findings	Reference
CRP	increased, median 6.5 mg/L – “low grade inflammation”, discriminates severity group	(6)
	higher pretransplant levels are predictive for cGvHD development	(99)
ESR	no significant association	(6)
WBC	no significant association with cGvHD, associated with decreased survival	(6)
C3 and C4 components of complement	increased, related to sclerotic changes, C3 discriminates severity group	(6, 123)
Total complement	no significant association	(6)
Coagulation		
VWF	increased compared to non-cGvHD post alloHSCT patients, associated with more active cGvHD	(108–110)
FVIII	increased compared to non-cGvHD post alloHSCT patients	(110)
Platelets	increased, correlates with more activity and fibrotic manifestations	(6, 123, 124)
	decreased – poor outcome	(117)
Platelet microparticles	increased	(125)
ADAMTS-13	slightly decreased, possible due to cytokine effect	(126)
Haptoglobin	if increased, predictive of cGvHD development	(127)
Proteomics		
sBAFF	increased, diagnostic, prognostic, correlates to activity and response to corticosteroids	(121, 128–130)
CXCL 9	increased	(121, 128, 129)
CXCL 10	increased	(121, 128)
CCL 15	increased	(128)
MMP3 (Matrix metalloproteinase 3)	increased, diagnostic for bronchiolitis obliterans syndrome	(128, 131)
CD163	increased, predictive	(128)
Other		
Ferritin	no significant association, correlates to use of systemic immunosuppression therapy, predictive for outcome	(6, 132)
Albumin	decreased, connected to activity	(6)
Circulating endothelial cell count	decreased, associated to sclerodermatous form of cGvHD	(133)

cGvHD, chronic graft-versus-host disease; CRP, c-reactive protein; ESR, erythrocyte sedimentation rate; WBC, white blood cells count; VWF, von Willebrand factor; FVIII, factor VIII; ADAMTs-19, a disintegrin and metalloproteinase with a thrombospondin type 1 motif, member 13; sBAFF, soluble B-cell activating factor; CXCL 9, chemokine (C-X-C motif) ligand 9; CXCL 10, chemokine (C-X-C motif) ligand 10; CCL 15, Chemokine (C-C motif) ligand 15; MMP3, matrix metalloproteinase 3; CD163, cluster of differentiation 163.

## Discussion

Elucidation of immunological processes in the recovery period following alloHSCT remains challenging, especially in context of aGvHD and cGvHD. It is well established that endothelial activation and injury enhance the occurrence of aGvHD (134). Furthermore, an increasing number of scientists advocate the existence of an endothelial form of GvHD (135). Pioneers of this thinking, Tichelli and Gratwohl, supported the hypothesis of the vascular endothelium as a target of GvHD-mediated immune responses, which were previously described by Biedermann and colleagues (108). In addition, there is also *in vitro* evidence that some of the endothelium targeting immunological effector cells are in fact endothelial-, not just allo-specific (136, 137). Moreover, Tichelli and Gratwohl proposed that vascular endothelial syndromes should be considered as different forms of aGvHD which are reflected by organ-related injury. Another support to this opinion was given by connecting endothelial damage to steroid-refractory aGvHD (138). More recently publications demonstrate a reduction of aGvHD severity by experimentally using the endothelium-protecting agents sildenafil and defibrotide (139–141). Of note, defibrotide can also reduce the allogenicity of endothelial cells towards CD8+ T cells (142).

As for cGvHD, although it is characterized by impaired immune tolerance mechanisms affecting innate and adaptive immunity, elevated levels of inflammation and endothelial injury are also present, albeit less well elaborated so far (6, 108). Although the role of endothelial injury in cGvHD biology is not fully elucidated yet, it is clear that disease activity correlates with activation of the endothelium. Such activation cannot be attributed to acute conditioning toxicity or engraftment, considering the long period of time passing between these events and cGvHD diagnosis. Taking into consideration the proportional increase of VWF in relation to cGvHD activity and severity, the most probable reason for endothelial activation must be immune-related mechanisms. It was proposed earlier that endothelial cells of small vessels can act as antigen presenting cells (APC), even in the absence of professional APC (143). Activated endothelial cells (EC) show increased surface histocompatibility antigens and co-stimulatory molecules, rendering them more susceptible to a direct immune attack by alloreactive donor T cells. The resulting EC activation and injury can facilitate the passage of donor-derived cellular and soluble effectors from the blood and into recipient tissues (5). Biedermann et al. described infiltration of alloreactive cytotoxic T lymphocytes in the upper dermis of cGvHD patient with sclerotic form of skin involvement and subsequent rarefaction of small vessels, which argues in favor of this observation (108). Moreover, it has been suggested that neovascularization inhibition supported by activated donor T cells may contribute to a graft-versus-leukemia (GvL) effect, which can explain the protective role of active GvHD in prevention of tumor relapse (144). Another investigation showed partial reconstitution of microvessels among long-term survivors after alloHSCT (median follow-up was 17 years in this study), with complete recovery of microvascular remodeling after resolution of skin cGvHD (145). In an interesting study performed by Willemze et al. percentage of EC chimerism was higher when measured over a longer period of time after alloHSCT (146). It can be concluded that microvascular loss and resultant tissue ischemia may contribute to target organ fibrosis characteristic of cGvHD and that the effect may be reversible if treated on time, pointing to a possible role for donor derived EC in repair of damaged blood vessels. Tichelli and Gratwohl additionally suggested a causative link between inflammation, endothelial injury and the increased risk of cardiovascular diseases reported among long-term alloHSCT survivors. They actually used a simple and logical explanation, connecting an accelerated atherosclerosis to endothelial injury which is caused by persistent vascular inflammation and provoked by immunological mechanisms of cGvHD (135). Considering all the facts, endothelium-protecting agents could significantly diminish this vicious circle of chronic inflammation, endothelial damage, and ischemia, and for their possible beneficial effect, low side-effect profile and preservation of GVL effect, their usage should definitely be taken into consideration for future treatment strategies in cGvHD. However, there are some limitations of this concept: there is lack of markers that differentiate between endothelial damage and acute phase reaction well, as well as lack of drugs that are endothelial protective and that are tested in sufficiently large studies to prove that endothelial protection is a goal that can be achieved.

To conclude, in search for much needed biomarkers of cGvHD for clinical use, this review draws attention to acute phase reactants that may be associated with cGvHD activity and severity of cGvHD organ involvement. As non-invasive, readily available, and cost-efficient markers of endothelial activation, VWF and FVIII could provide valuable insight into cGvHD activity. Their elevation has a promising biomarker potential for an early diagnosis and as surrogate for activity of the disease. This concept is further underpinned by their proven good prognostic value in chronic autoimmune diseases clinically resembling cGvHD. In addition to the aforementioned factors it may also be helpful to assess endothelium-derived microvesicles for endothelial injury and procoagulant activity (147). Still, the influence of patient-related parameters should definitely be taken into consideration, and for that reason further prospective studies addressing this issue and the precise role of endothelial injury in cGvHD in general are needed.

## Author Contributions

AL: drafting of the manuscript, acquisition and interpretation of data. HG: interpretation of data, critical revision of the manuscript for important intellectual content. DW: interpretation of data, critical revision of the manuscript for important intellectual content. GE: interpretation of data, critical revision of the manuscript for important intellectual content. SP: interpretation of data, critical revision of the manuscript for important intellectual content. DP: designed and coordinated the preparation of the manuscript, interpretation of data, critical revision of the manuscript for important intellectual content. All authors contributed to the article and approved the submitted version.

## Funding

This work was supported by European Cooperation in Science & Technology under the COST Action CA17138 (Integrated European Network on Chronic Graft Versus Host Disease) and by the Croatian Science Foundation Project IP-2016-06-8046 entitled “New biomarkers for chronic Graft-versus-Host disease”.

## Disclaimer

The opinions expressed here are those of the authors and do not represent the official position of the National Institutes of Health or the US Government.

## Conflict of Interest

The authors declare that the research was conducted in the absence of any commercial or financial relationships that could be construed as a potential conflict of interest.
